# Relationship between Having a Home Doctor and Outpatient Utilization

**DOI:** 10.2188/jea.11.160

**Published:** 2007-11-30

**Authors:** Seiji Bito, Shunichi Fukuhara, Martin F Shapiro, Hideki Hashimoto, Kiyoshi Kurokawa

**Affiliations:** 1 Department of General Internal Medicine, National Tokyo Medical Center.; 2 Department of Epidemiology and Health Care Research, Kyoto University Graduate School of Medicine and Public Health.; 3 Department of Medicine, University of California, Los Angeles.; 4 Department of Public Health, School of Medicine, Teikyo University.; 5 School of Medicine, Tokai University.

**Keywords:** resource utilization, equity in health services, home doctor, health system in Japan

## Abstract

While universal insurance coverage should eliminate or substantially reduce financial and certain structural barriers to medical care, inequity in utilization of care may continue to exist. We conducted a questionnaire survey of a national random sample of 4500 Japanese age16 or over in October, 1995. Separate analyses were conducted to predict the physician visit rates for the entire respondents (N=3395) and for those with chronic conditions (N=777). Forty-three percent of the total subjects reported an ambulatory physician visit within the past three months. About 17% of subjects with one chronic condition and 14% of those with two or more chronic conditions did not have any physician visits within recent three months. The regression model demonstrated that having a home doctor, as well as comorbidity and perceived health status, is significantly associated with outpatient visit both among all subjects (p< 0.0001) and among those with chronic conditions (p< 0.01). The Japanese health system still has unevenness in outpatient resource utilization. This mainly pertains to whether they have their own regular physician. The failure of some persons with chronic diseases to be seen requires further investigation.

## INTRODUCTION

To achieve better equity in access to medical care is an important issue for the health care systems in many countries^1^^-^^3^^)^. While many factors affect access, the extent and characteristics of health insurance coverage is one of the most important dimensions relevant to patterns of utilization^4^^-^^12^^)^.

Like some European industrialized countries, Japan has a universal health insurance system. This system is fee-for-service and strictly regulated by the government so that health care consumers can receive equal financial coverage^13^^, ^^14^^)^. Consumers under 70 years of age usually pay 10% to 30% of total medical charges to providers directly, and the rest is paid by the social or national insurance program^13^^, ^^14^^)^. This arrangement should, at least in theory, reduce financial barriers to utilization of basic health resources^14^^, ^^15^^)^.

Yet, some characteristics of Japanese health system which are not related to financial barriers may militate against health resource utilization^16^^, ^^17^^)^. One problem is the difficulty in establishing a continuing relationship with a primary care doctor. Unlike the system in the United Kingdom, Japan has no official system assigning general practitioners to patients. Though free to choose any physician, Japanese health consumers must take initiative in establishing continuing relationships with primary health providers^18^^)^. In spite of extensive commentary on the Japanese health care system and on resource utilization in it^19^^)^, there have been very few empirical studies of the extent of actual and potential barriers to equitable resource use in this system.

This study was undertaken to assess the factors influencing ambulatory resource utilization in Japan, in order to identify areas in which interventions are needed to improve patterns of utilization. The purpose of this study is to explore whether outpatient care utilization is related to patients’ health belief, subjective health perceptions, and potential accesibility such as having their own home doctors.

## MATERIALS AND METHODS

### Conceptual Framework, for Ambulatory Resource Utilization

When conducting this analysis, we employed Andersen’s widely used theoretical model of health access^20^^)^. According to his model, there is two main dimensions affecting a population’s utilization of health resources: the health environment and the population characteristics. The health environment is defined to include both the medical technologies available for patient care and the financial arrangements through which care is financed.

**Figure 1.**
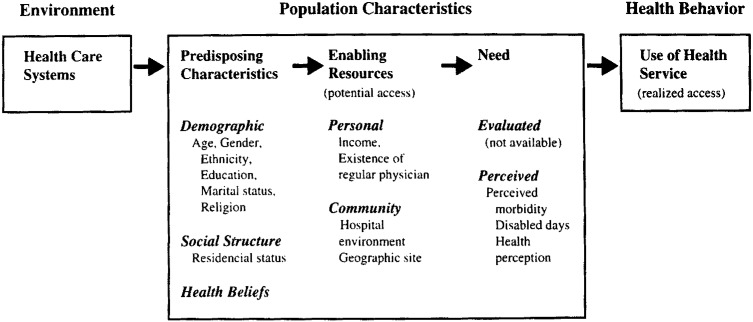
Conceptual framework for health services utilization.

Population characteristics are grouped into three categories: predisposing characteristics, enabling resources, and need. Predisposing characteristics include demographic variables and health beliefs. Enabling resources pertain to insurance status, socio-economic status and geographic accessibility. Need is usually determined by severity of disease and the provider’s or individual’s own estimation of health status. Figure 1 presents this theoretical model of resource utilization.

Andersen articulated the relationship between health care utilization and access to health care. Utilization of health resources is defined as “realized access” in contrast to the “potential access” of enabling resources present. He explains that equitable realized access occurs when demographic and need variables account for most of the variance in utilization, and inequitable realized access occurs when social structure, health beliefs, and enabling resources determine who gets medical care^20^^)^. In this context, we were interested in determining the extent to which social structure, health beliefs and enabling factors within the population influence utilization of health services in Japan.

### Subjects and Data Collection

This cross-sectional study was based on a self-administered questionnaire survey conducted from October to November 1995. The study enrolled a stratified random sample of all age 16 years or older people living in Japan. The country was divided into 45 strata according to 9 geographic regions and 5 levels of population size. We then randomly selected and allocated 300 clusters in strata, which were weighted using census data. Next, 15 people on average were randomly sampled from each cluster. When we chose specific individuals for sampling, we used residential records of each selected region. In this way, 4,500 subjects were randomly sampled for this survey. The questionnaire asked about their health behaviors, perceptions and preferences. A self-addressed envelope was hand-delivered with a copy of the questionnaire, which took an estimated 30 minutes to complete. When we distributed the questionnaires, we stated to the respondents that the collected data must be treated anonymously and the respondents’ privacy should be protected. Persons who returned the questionnaires were given a telephone calling card valued 1,000 yen at completion.

### Questionnaire and Variable Definitions

We collected data on predisposing, enabling and need factors predicting health resource utilization according to the theorized model (Figure 1). Predisposing characteristics pertain to a population’s personal demographics, social structure, and health beliefs. Age, gender and marital status were requested demographic information. Level of education variables and whether an individual lives alone measured social structure. Health belief variables often have been excluded in previous studies because of difficulties measuring them^21^^-^^23^^)^. In this survey, we used a nine-item version of the health locus of control (LOC) belief scale, which includes three dimensions of LOC preferences: internal control, control by powerful others such as physicians or other health professionals, and control that is not localized to either the individual or other people (“control by chance”)^24^^)^. The Japanese version of the LOC scale is introduced in a previous study^25^^)^. Each dimension of the LOC scale has three questions and is scored into a 0-100-point scale by the Likert method.

Enabling resources usually included financial status, insurance status, geographical accessibility and relationship with physicians or health facilities. We asked the respondents’ annual family income to measure their financial status. The residential site variable was indicated by the zip code where each subject lived. Additionally, we asked whether the subject had a home doctor (KAKARITSUKE-I) whom usually provided his or her care.

To measure health consumers’ needs, we surveyed subjects about their number of specific chronic diseases, total annual days unable to work due to disability, and subjective health perceptions. A respondent’s report of specific chronic conditions was divided into 14 categories 6 of which were serious and usually need continuous care ; each category about which they were questioned included chronic conditions of which the individual is likely to have been aware the following 6 disease categories; hypertension, diabetes, cerebrovascular disease, miocardial infarction, angina, and heart failure or cardiomegaly. Self-report answers were multiple choice. Respondents were asked whether they had days off from work due to disability and, if so, the number in the previous year. We also used the general health perception subscale of the Medical Outcome Study (MOS) Short-Form 36-item health survey questionnaire (SF-36)^26^^, ^^27^^)^, that consists of five-item scale questions and was transformed to a 0-100-point score.

Concerning resource utilization, we focused on use of outpatient resources excluding dental care, acupuncture and other alternative medical therapies. Respondents were asked whether they actually had visited an outpatient clinic or physician’s office in the past three months, and to report the actual number of visits to the clinic. We did not ask style type of patient services. The exact question was “Have you been to a clinic or a hospital ambulatory office in the past three months? If yes, how many times?”

### Data Analysis

Ambulatory utilization was categorized into four groups: no visits, 1-3 visits, 4-6 visits, and 7 or more visits and was analyzed by age group strata and by number of comorbidities.

To explore the predictors of realized access to ambulatory care services, we used multivariate analysis. First, logistic regression analysis was employed to predict whether a person received any ambulatory services during the previous three months. Based on the conceptual model (Figure 1), we developed a regression model using the following formula:

$$\begin{array}{rl} & \text{Existence of any physician visits (yes, no)}\\ & \;=\text{f }\{\text{Intercept}+\text{Demographics (age, gender, marital status)}\\ & \;+\text{Social Structure (residential status, education)}+\text{Locus}\\ & \;\text{of Control}+\text{Enabling Resources (family income, geo-}\\ & \;\text{graphic site, existence of home doctor)}+\text{Need (number of}\\ & \;\text{comorbidities, annual days unable to work, general health}\\ & \;\text{perception)}\}. \end{array}$$

For developing the logistic model, all of the categorical variables of the predictors were designed to function as dummy variables: age (16-34, 35-49, 50-59, 60-69, 70 and over), gender (male, female), marital status (married, single, widowed or divorced), education (high school, college, above university level), geographic site (rural area, city population of <150,000, city population of >150,000), LOC (high [50%tile score or over in total subjects] vs. low [below 50%tile score in total population] LOC: internal, powerful others and chance), income (<$30,000 per year, $30,000-$70,000 per year, >$70,000 per year), residential status (living alone, living with someone), existence of home doctor (have no home doctor, have home doctor), number of serious chronic diseases (have no chronic disease, have one chronic disease, have two or more chronic diseases), total days unable to work due to disability (no days disabled, 1-9 days disabled, 10 or more days disabled), and general health perception subscale score in the SF-36 (low [0-39] score group, middle [40-69] score group, high [70 or over] score group). Because the SF-36 score is originally a continuous variable, we categorized it into these three levels according to one- and three-quarter level. We computed odds ratios and 95% confidence intervals for the independent variables. The analysis was conducted not only among all subjects available but also separately for those subject who had at least one serious chronic disease. Of the 3,368 answers, 95 were not included in the model because of missing data of the set of the independent variables.

Second, we evaluated the relationships of these predictors to number of ambulatory visits among the subjects with serious chronic diseases, to identify factors explaining potential underutilization in care or lack of continuity. In the specific analytic process, the subjects were included in the analysis if they answer “yes” to one or more chronic disease categories in the comorbidity question. We employed analysis of variance to compare adjusted mean frequencies of ambulatory use. The independent variable model also included age group, gender, marital status, supporting family, residential site, LOC, family income, existence of regular physician, number of chronic diseases, total annual days unable to work, and general health perception as categorical variables with frequency of ambulatory resource use as a dependent variable. Variable categories of each independent variable were the same as that in the logistic model. Least square means for all significant variables were presented.

Because our purpose in this analysis was to identify factors that are associated with underutilization of resources, we set a “ceiling” value of frequent use in this study. We assumed that visiting ambulatory physicians more than once a week on average does not meaningfully improve access compared to visiting physicians once a week. Additionally, small numbers of outliers in the dependent variable could affect linear equations of the predicting model, and thereby distort the linear relationship between variable^28^^)^. We therefore set “15 visits within three months” as the ceiling value.

## RESULTS

Of the 4,500 subjects, 3,413 of all subjects sampled returned the survey booklets of which 3,395 were usable (response rate was 75.6%); 3,368 answered the question about ambulatory care utilization. Characteristics of the respondents are presented in Table 1. Only about half had their own home doctor and approximately 13% of the subjects had at least one serious chronic disease. Though demographic and socio-economic characteristics were quite similar between all sampled and those with chronic conditions, about 18% of the subjects with chronic conditions including hypertension, diabetes, cerebrovascular disease and chronic heart diseases did not have a home doctor. As expected, the general health perception score in SF-36 was much lower among subjects having chronic diseases than among total subjects (p<0.001).

**Table 1.** Characteristics of the respondents.

Characteristics	All subjects (N = 3368)	Subjects with chronic diseases (N = 429)
Age (years)		
Mean (SD)	46.2 (16.4)	61.7 (10.8)
Range	16 - 93	16 - 93
Gender, % (No.)		
Female		
Schooling, % (No.)		
Below graduated from high school	21.1 (702)	43.3 (182)
Graduated from high school	46.5 (1543)	41.0 (172)
Some college or technical school	15.9 (529)	6.2 (26)
Above graduated from college	16.5 (548)	9.5 (40)
Marital Status, % (No.)		
Single	19.2 (642)	2.6 (11)
Married	73.6 (2462)	83.8 (357)
Divorced / separated	2.2 (73)	2.1 (9)
Widowed	5.1 (172)	11.5 (49)
Residencial Family Status, % (No.)		
Living alone	5.1 (171)	6.1 (26)
Living with someone	94.9 (3197)	93.9 (403)
Residencial Site, % (No.)		
Rural area	23.7 (799)	26.3 (113)
Mid city (less than 150,000 of population)	26.2 (883)	24.8 (106)
Large city (more than 150,000 of population)	50.1 (1686)	48.9 (210)
Family Income, % (No.)		
Under $30,000 per year	13.5 (422)	22.8 (92)
$30,000 - $70,000 per year	47.6 (1483)	44.9 (181)
Over $70,000 per year	38.9 (1213)	32.2 (130)
Health locus of control scale, means (SD)		
LOC / internal	71.4 (16.1)	75.2 (15.3)
LOC / powerful others	64.6 (17.1)	75.0 (15.3)
LOC / chance	38.8 (20.7)	41.8 (22.7)
Existence of regular physician, % (No.)		
Have no regular physician	45.9 (1554)	18.4 (79)
Have at least one regular physician	54.1 (1817)	81.6 (350)
Number of chronic diseases, % (No.)		
Do not have any chronic disease	87.3 (2939)	
Have one chronic disease	10.3 (346)	80.7 (346)
Have two or more	2.5 (83)	19.3 (83)
Total annual disabled days for work, % (No.)		
no disabled days	70.9 (2354)	70.9 (290)
one to nine disabled days	17.8 (592)	9.8 (40)
ten or more disabled days	11.2 (373)	19.3 (79)
General health perception score of SF-36, means (SD)	65.0 (19.6)	51.4 (19.9)

Table 2 shows ambulatory care utilization by age group and by number of chronic diseases. Use of any ambulatory care increased with age. While 43% of all subjects reported at least one ambulatory physician visit within the past three months, the rate of utilization of ambulatory care services was much higher among the elderly aged 70 or over. Almost three of four persons without chronic diseases had had no visits in the last 3 months. Among those with one or more chronic diseases, most, but not all did use some care; 17% of those with one chronic disease and 10% of those with two or more chronic diseases did not have any physician visits within the recent three months.

**Table 2.** Utilization ambulatory care and hospital admissions by age and chronic conditions.


Age *	No visits past 3 month, No. (%)	1 - 3 visits past 3 months, No. (%)	4 -6 visits past 3 months, No. (%)	More than 7 past 3 months, No. (%)

16 - 34	603 (66.7)	210 (23.2)	44 ( 4.9)	47 ( 5.2)
35 - 49	625 (63.1)	236 (23.8)	70 ( 7.1)	60 ( 6.1)
50 - 59	376 (55.3)	170 (25.0)	78 (11.5)	56 ( 8.2)
60 - 69	246 (44.7)	120 (21.8)	103 (18.7)	81 (14.7)
Over 70	83 (34.2)	44 (18.1)	56 (23.1)	60 (24.7)

Total	1933 (57.4)	780 (23.2)	351 (10.4)	304 ( 9.0)

* There is a significant differences in frequency (p<0.001 chi-square test).				


Chronic conditions**	No visits past 3 month, No. (%)	1 - 3 visits past 3 months, No. (%)	4 -6 visits past 3 months, No. (%)	More than 7 past 3 months, No. (%)

No chronic disease	1865 (63.5)	644 (21.9)	223 ( 7.6)	207 ( 7.0)
Have one chronic disease	59 (17.1)	113 (32.7)	102 (29.5)	72 (20.8)
Have two or more chronic diseases	9 (10.8)	23 (27.7)	26 (31.3)	25 (30.1)

Total	1933 (57.4)	780 (23.2)	351 (10.4)	304 ( 9.0)

** There is a significant differences in frequency (p<0.001 chi-square test).				

### Factors Related to Whether or Not Subjects Used Ambulatory Care Services within the Three Months: All Subjects and Those with Comorbid Conditions (Table 3)

**Table 3.** Associations of its predictors with whether people had access to ambulatory physician visits during previous three months : The logistic model.

	All subjects (N = 3349)	Among the subjects who have one or more chronic diseases (N = 420)

Variable	Adjusted Odds Ratio (95% CI)	
Age		
16 - 34	0.97 (0.76-1.22)	0.52 (0.05-5.37)
35 - 49 (reference = r)	1	1
50 - 59	1.07 (0.85-1.35)	0.74 (0.25-2.17)
60 - 69	1.17 (0.90-1.52)	0.72 (0.25-2.05)
Over 70	1.69 (1.10-2.30)	0.9 (0.28-2.91)
Gender		
Male (r)	1	1
Female	1.48 (1.01-2.17)	0.8 (0.43-1.50)
Schooling		
Below some college level(r)	1	1
Some college or technical school	0.99 (0.79-1.25)	1.41 (0.37-5.35)
Graduate college or above	1.14 (0.91-1.43)	1.59 (0.59-8.12)
Marital status		
Single, devorced or widowed (r)	1	1
Married	1.09 (0.88-1.36)	1.46 (0.58-3.62)
Residensial site		
Rural	0.76 (0.61-0.95)	0.52 (0.24-1.16)
Mid city (less than 150,000 of population) (r)	1	1
Large city (more than 150,000 of population)	1.03 (0.85-1.24)	1.11 (0.54-2.29)
Supporting family member		
Living with one or more family member (r)	1	1
Living alone	0.99 (0.65-1.49)	3.76 (0.66-21.52)
Family income		
Below $30,000 / year	1.05 (0.80-1.38)	0.78 (0.37-1.68)
$30,000 - $70,000 / year (r)	1	1
Over $70,000 / year	1.01 (0.84-1.21)	0.67 (0.35-1.31)
Existence of regular physician		
have no regular physician (r)	1	1
have at least one regular physician	2.11 (1.80-2.49)	2.24 (1.17-4.31)
Number of chronic disease ^		
have no chronic disease (r)	1	NA
have one chronic disease	5.62 (4.08-7.74)	1
have two or more chronic diseases	6.29 (3.01-13.12)	1.6 (0.70-3.64)
Annual disabled days		
no disabled days (r)	1	1
one to nine annual disabled days	2.2 (1.7 -2.70)	2.85 (0.80-10.15)
ten or over annual disabled days	4.9 (3.70-6.53)	2.86 (1.08- 7.59)
General health perception score of SF-36¶		
Low (below 39 points) score group	1.67 (1.19-2.33)	0.53 (0.24-1.16)
Middle (40-69 points) score group (r)	1	1
High (above 70 points) score group	0.57 (0.48-0.67)	0.42 (0.21-0.82)
Locus of control scale (low point group as r)¶		
High internal locus of control (points or over)	0.85 (0.72-1.00)	0.73 (0.39-1.37)
High others locus of control (points or over)	1.52 (1.26-1.82)	0.93 (0.50-1.74)
High chance locus of control (points or over)	0.84 (0.71-0.99)	0.76 (0.42-1.37)

+ The logistic model had ROC area = 0.78.

++ The logistic model had ROC area = 0.73.

^ In the analysis among the subjects who have more than one chronic disease, we put single disease group as a reference, and compared with poly-disease group.

¶ Cronbach’s alfa values of the general health perception score of the SF-36 and each subscale of the locus of control scale were 0.86, 0.68, 0.59, 0.71, respectively.

The logistic regression model identified some significant predictors of whether the subjects had an ambulatory clinic visit during the previous three months. In terms of demographic characteristics, subjects aged 70 or over were more likely to visit a physician (OR = 1.69; 95%CI:1.10-2.30) and those who wee living in rural area were less likely to visit (OR = 0.76; 95%CI:0.61-0.95). Women were more likely to visit than men (OR = 1.48; 95%CI:1.01-2.17). No other demographic and social structure variable including marital status, educational level and family annual income was significantly association with having an outpatient visit. Those who had their own home doctor were more likely to make outpatient visits than those lacking such a provider were (OR = 2.11; 95%CI:1.80-2.49). Respondents who were more likely to report an ambulatory visit had high scores for a locus of control on “powerful others” (OR = 1.52; 95%CI:1.26-1.82). Subjects with two or more serious chronic disease categories were much more likely to have outpatient visits than those with one chronic disease (OR=5.62; 95% CI:4.08-7.74 for one chronic disease; OR=6.29; 95% CI:3.01-13.12 for two or more chronic diseases). Total annual days missing work due to disability and scores on the general health perception scale also were significantly associated with having a visit.

In the analysis of the respondents having chronic diseases, people with higher general health perception scores were less likely (OR=0.42; 95%:CI 0.21-0.82), and those who had ten or more disabled days in a year were more likely (OR=2.86; 95% CI:1.08-7.59) to have made outpatient visits than the respective reference groups. The most significant predictor of whether the respondents with chronic diseases had an outpatient visit was whether they had their own regular physician (OR=2.24; 95% CI:1.17-4.31).

### Factors associated with Number of Ambulatory Visits among Subjects with Chronic Conditions: Multivariate Model (Table 4)

**Table 4.** Comparison of least square mean in number of utilization in 3 months : among subjects with chronic diseases.

Variable	Adjusted Odds Ratio (95% CI)	
By Age Group		
16-34	3.02	(-0.82-6.86)
35-49	4.72	( 3.22-6.22)
50-59	4.77	( 3.47-6.07)
60-69	5.49	( 4.27-6.71)
Over 70	6.16	( 4.81-7.51)
By Existence of Regular physician		
Have no regular physician	4.55	( 3.08-6.02)
Have at least one regular physician	5.11	( 3.82-6.40)
By General health perceptions score of SF-36*		
Low (0-39) score group	5.45	( 4.03-6.85)
Middle (40-69) score group	5.3	( 3.93-6.67)
High (over 70) score group	3.75	( 2.14-5.36)

The model included the following variables: gender (female, male); marital status (married, not married); annual family income (under $30,000, $30,000 - $70,000, over $70,000); residencial site (urban, mid-city, rural or small city); residencial status (living alone, living with someone); number of chronic diseases (have one chronic disease, have two or more diseases), annual disabled days (no disabled days, one to nine disabled days, ten or over disabled days), locus of control (high vs low self LOC/internal, high vs low LOC/others, high vs low LOC/chance; all these variables did not have significant difference.

* : p < 0.05

Least squares regression revealed that general health perception score (p<0.05) were significantly related to the number of ambulatory visits within the three months. Age group, annual family income and number of chronic diseases were not associated with the frequency of outpatient visits.

## DISCUSSION

This study examined factors associated with Japanese residents’ utilization of ambulatory care under the conditions of universal health insurance. We examined both whether respondents had a visit in a 3-month period and how many times they were seen. Older subjects were more likely to report any visit and also visited more frequently than younger subjects, a finding consistent with the higher level of morbidity in the older age group. We found that substantial minority of subjects with chronic diseases did not report ambulatory visits in the study period. Although the study methodology did not permit detailed analysis of these cases, this subset of the population are potentially at higher risk for not having sufficient continuity and use of care in relation to their needs.

The multivariate analyses showed that ambulatory visit use was affected by factors that reflect need for care, such as number of chronic diseases, days in a year unable to work due to disability and general perceived health. Since need should drive utilization, these are reasonable findings. Andersen has argued that health beliefs of people are moderately changeable while demographic variable and social structure are hardly changeable^20^^)^. At the same time, our analysis demonstrates that differences in health locus of control preferences had a significant relationship with outpatient resource use. Strategies to change health consumers’ attitudes and beliefs may be needed to increase further the appropriateness in the pattern of utilization of care. Access to outpatient care of those living in rural area was smaller than that among more urbanized citizens. Geographical barriers to health access may exist even in Japan where most residents have few traffic problems^29^^)^. We rather have to consider that population strategy of health services for rural area is not enough. Some educational programs enhancing health service use may be needed.

One noteworthy result in this national survey was that social status and enabling resources such as annual family income did not show as significant variables associating with ambulatory access and its frequency. In Japanese social insurance system, insurance certificates are distributed to all individual workers, employers and elderly people over age 70^13^^, ^^30^^)^. Very few people who do not have social insurance are under welfare program that covers all medical fees^13^^, ^^30^^)^. Outpatient care services delivered for the elderly was free while the insured usually pay 10% of total fee, and their family pay up to 30% for received care as of 1995. For main reason why social structure and financial factors does not affect health utilization in Japan, Japanese ordinary people have smaller economic variances then expenditures for 10-30% of medical costs does not reach the threshold which makes differences in ambulatory health services use.

Perhaps of greatest relevance to Japanese health policy, having a continuous relationship with a health care provider was an important predictor of utilization. Even as universal health coverage eliminates some financial barriers against health services use, absence of a regular physician on which the consumer relies for his or her health care could be an important barrier to achieving equitable outpatient resource utilization. Some people with health problems may be reluctant to visit a clinic or doctor’s office because the medical facility is regarded as hostile or intimidating, because of bureaucratic nonfinancial barriers to being seen.

Having a regular physician was similarly associated with use among those with chronic diseases. It is reasonable that those with chronic diseases more often had regular providers. On the other hand, there were no significant associations between age, gender, marital status and even annual income variables with whether those with chronic conditions visited a physician. Our findings did not identify any income-related barriers to obtaining at least some care for Japanese health consumers with need for ambulatory care^31^^)^. However, Japan needs assess the extent to which factors such as these are inhibiting use of care, particularly among persons with multiple chronic diseases who are being seen infrequently.

Frequency of physician visits was influenced only by perceived health status. While patients have substantial influence over whether they are seen at all, the number of follow-up visits, particularly with one provider, are usually physician-driven^32^^)^. Concerning general health perception and frequency of outpatient use, we can assume that low health perception of health consumers enhance demand of seeing physicians that may release their anxiety. Though demand-oriented utilization is reasonable in health service model, excessive pessimism about subjective health may cause overuse of health services. For physicians, supportive communications enhancing patients’ health perception make good patient-physician relationship and may eliminate more appropriate outpatient services use.

The main mission of a universal health coverage system is to provide the population with equal access to health services. It should result in all people who need care getting in on a more or less equal basis. Our study has demonstrated that the Japanese national universal health insurance system has not eliminated disparities in resource use. Some people who are likely to need care by health professionals may not be obtaining it, either because of the deterrent effect of copayments, or because they do not have a regular provider.

Japanese people are virtually unrestricted in their choice of doctors and hospitals, but such a free-access system may potentially make continuity of care difficult and diminish appropriate utilization^33^^)^. Fostering ongoing relationships between patients and primary care practitioners has become more difficult in recent years as ambulatory practices have moved to larger hospitals. Programs enhancing more stability in patient-physician relationships in a community are needed. Possible strategies include highlighting primary care, its content and value in medical training programs.

This study has several major limitations. First, since frequency of utilization was self-reported, the validity of the responses may be limited^34^^)^. Outpatient resource use within a span of three months as the dependent variable may not be adequate for certain especially healthy people. Analyses that take account of annual utilization may be more appropriate for understanding adequacy of use among the general population and, perhaps, among some persons with major chronic diseases whose need for ongoing surveillance is low (such as well-controlled hypertension).

Second, we did not measure out-of-pocket costs. Some different co-payment rate exists in Japanese national or social insurance program according to an insurer’s working status. Moreover, some Japanese actually get reimbursed from a private insurance company for part of the remaining cost of their care. We did not ask whether the subjects have this optional financial support which clearly will affect the extent of financial barriers to service use. Third, measurement of variables was not fully adequate. Although we used measures for subjects’ health perceptions and the existence of comorbidities, for example, we did not assess episodes of acute illness or measure directly the severity of the chronic diseases. In addition, definition of “having chronic diseases” is completely dependent on self report and it may not have strong validity for measure though this variable have an important role in this analysis. It would have been helpful to have collected data about type of facility in which care was delivered, physician specialty, respondents’ prior use of services and included them in our model to waive weak points of cross-sectional study. Though type of health source and type of physician is not different across the social strata in Japan, health access and its frequency should be different across type of clinics. In Japanese free-access system, as we related before, patients can freely choose any type of clinic and services fee is the same for same contents of services at any type of sources. In general, many people have a bigger demand to go for ambulatory resources in large hospitals than that for small clinic. Therefore, physicians working at clinic in the large hospitals commonly have incentives to limit frequency of patients access to their services. Ethnicity variable should also have been asked. Physicians at hospital are employed with fixed salary and little incentives to see many patients. There are indeed some minority ethnic groups in Japan, including Korean Japanese and Ainu an aboriginal people in the north of the country. Because such populations are so small, this variable is usually omitted in Japanese surveys. Moreover, we did not have detailed information about the patient-physician relationship and how subjects utilized outpatient care. For example, we cannot distinguish the purpose of the physician visits and did not determine who initiated the visit.

The biggest concern for our study is how we should interpret the associations between having regular physicians and health utilization. Having a regular source of care could well have been stimulated by use. In the cross sectional study, we definitely cannot reject the hypothesis that the perception of a regular source of care is not a predictor but a result of resource use, even though we got same result analyzed with sample who have serious chronic diseases.

In summary, our study has revealed that inequity still exits in utilization of outpatient care in Japan, even though the Japanese health system provides universal insurance coverage. Further study is needed to assess the causes of these disparities and the extent to which lack of continuity with a regular providers, health insurance and patient preferences and attitudes are responsible for.
